# Supplementary material for: Is a 2D Nanostructured Surface Capable of Changing the Corrosion and Magnetic Properties of an Amorphous Alloy?
Source: Int J Mol Sci. 2023 Aug 29;24(17):13373. doi: 10.3390/ijms241713373 (PMC10488001; doi:10.3390/ijms241713373)
Supplement: Supplementary file 1 [file ijms-24-13373-s001.zip › ijms-2557937-supplementary.pdf]

International Journal of *Molecular Sciences*

MDPI

*Supplementary Materials*

# Is a 2D nanostructured surface capable of changing the corrosion and magnetic properties of an amorphous alloy?

**Irina Kuznetsova [1], Olga Lebedeva [1,*], Dmitry Kultin [1], Natalia Perova [2], Konstantin Kalmykov [1], Petr Chernavskii [1], Nikolai Perov [2], and Leonid Kustov [1,3,4,*]**

Department of Chemistry, Lomonosov Moscow State University, Moscow, 119991, Russia; kuznetsowair@yandex.ru (I.K.), lebedeva@general.chem.msu.ru (O.K.), dkultin@general.chem.msu.ru (D.K.), lmk@ioc.ac.ru (L.K.), chern5@inbox.ru (P.C.), kbkalmykov@mail.ru (K.K.)

[2] Faculty of Physics, Lomonosov Moscow State University, 119991 Moscow, Russia; perov@magn.ru (N.P.), perova.n@physics.msu.ru (Nt.P.)

[3] N.D. Zelinsky Institute of Organic Chemistry, Russian Academy of Sciences, Leninsky Prospect 47, Moscow, 119991, Russia

[4] Institute of Ecology and Engineering, National Science and Technology University "MISiS", Leninsky Prospect 4, Moscow, 119049, Russia

* Correspondence: lmk@ioc.ac.ru, lebedeva@general.chem.msu.ru

*Preparation method of the amorphous Co75Si15Fe5Cr4.5Al0.5 alloy*

The rotating-cylinder method has been applied for the preparation of the amorphous iron-chromium-silicon alloy in the form of a ribbon. The starting materials were Co, Si, Fe, Cr and Al (purity 99.95-99.99%). The amorphous Co75Si15Fe5Cr4.5Al0.5 alloy was prepared in an electric arc furnace in an argon atmosphere with the triple remelting procedure. Impinging a stream of molten alloy on the outer surface of a rotating cylinder in an argon atmosphere was used to convert the obtained crystalline sample to the amorphous state. The ribbon parameters were: width 7–10 mm, thickness 10 µm. The preparation conditions were determined by the necessity to obtain the material in the amorphous state. The amorphous alloy was obtained as a ribbon by high-speed quenching of the melt on a fast-moving substrate. The cooling rate was 106 K/s. The composition of the alloy was controlled by local micro-X-ray spectral analysis using an EVO-50 Zeiss scanning electron microscope EVO-50.

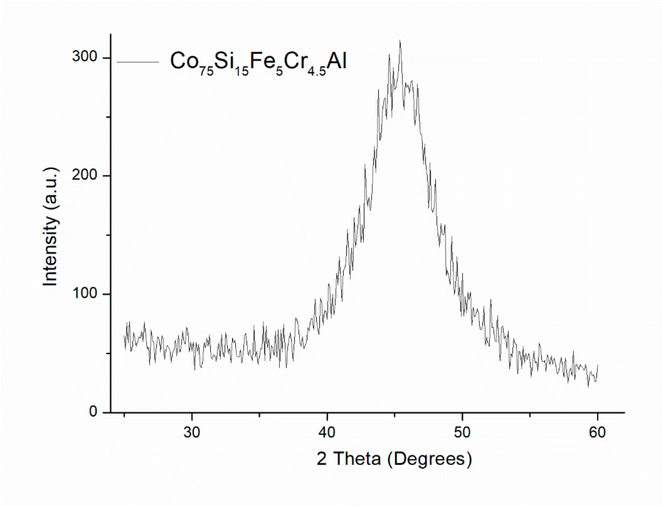


**Figure S1.** XRD pattern of the alloy sample 2 before annealing (700° C) (amorphous sample).

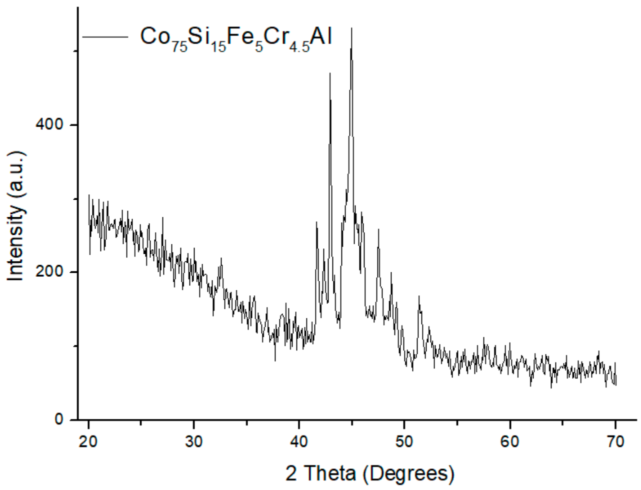


**Figure S2.** XRR pattern of the alloy sample 2 after annealing (700°C) (the sample underwent crystallization and stratified into two phases).

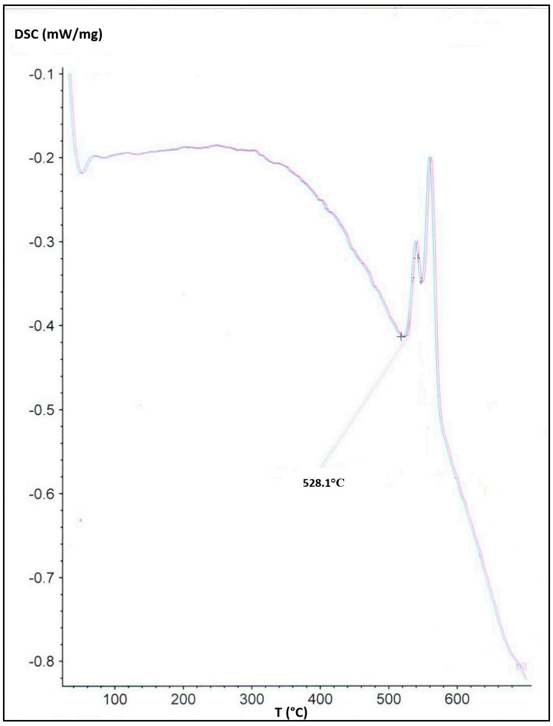


**Figure S3.** DSC curve for the alloy sample 2. The transition to the two-phase crystalline state is clearly visible.
